# Intratumoral Treatment with Chemotherapy and Immunotherapy for NSCLC with EBUS-TBNA 19G

**DOI:** 10.7150/jca.55322

**Published:** 2021-03-05

**Authors:** Paul Zarogoulidis, Wolfgang Hohenforst-Schmidt, Haidong Huang, Jun Zhou, Qin Wang, Xiangqi Wang, Ying Xia, Yinfeng Ding, Chong Bai, Christoforos Kosmidis, Konstantinos Sapalidis, Chrysanthi Sardeli, Kosmas Tsakiridis, Bojan Zaric, Tomi Kovacevic, Vladimir Stojsic, Tatjana Sarcev, Daliborka Bursac, Biljana Kukic, Sofia Baka, Evagelia Athanasiou, Dimitrios Hatzibougias, Electra Michalopoulou-Manoloutsiou, Savvas Petanidis, Dimitris Drougas, Konstantinos Drevelegas, Dimitris Paliouras, Nikolaos Barbetakis, Anastasios Vagionas, Lutz Freitag, Aimilios Lallas, Ioannis Boukovinas, Dimitris Petridis, Aris Ioannidis, Dimitris Matthaios, Konstantinos Romanidis, Chrisanthi Karapantzou

**Affiliations:** 1 3rd Department of Surgery, ``AHEPA`` University Hospital, Aristotle University of Thessaloniki, Medical School, Thessaloniki, Greece.; 2 Sana Clinic Group Franken, Department of Cardiology / Pulmonology / Intensive Care / Nephrology, ''Hof'' Clinics, University of Erlangen, Hof, Germany.; 3 Department of Respiratory and Critical Care Medicine, First Affiliated Hospital of Naval Medical University ( Changhai Hospital, Second Military Medical University), Shanghai, China.; 4 Department of Respiratory, Changzhou maternal and child health care hospital affiliated to Nanjing Medical University, Jiangsu Changzhou, China.; 5 Thoracic Surgery Department, ``Interbalkan`` European Medical Center, Thessaloniki, Greece.; 6 Faculty of Medicine, University of Novi Sad, Institute for Pulmonary Diseases of Vojvodina, Novi Sad, Serbia.; 7 Oncology Department, ``Interbalkan`` European Medical Center, Thessaloniki, Greece.; 8 Private Pathology Laboratory, "Microdiagnostics", Thessaloniki, Greece.; 9 Department of Pulmonology, I.M. Sechenov First Moscow State Medical University, Moscow, Russian Federation.; 10 Scientigraphy Department, "Bioclinic" Private Laboratory, Thessaloniki, Greece.; 11 Radiology Department, "Euromedica" Private Radiology Laboratory, Thessaloniki, Greece.; 12 Thoracic surgery Department, ``Theageneio`` Cancer Hospital, Thessaloniki, Greece.; 13 Oncology Department, General Hospital of Kavala, Kavala, Greece.; 14 Department of Pulmonology, University Hospital Zurich, Rämistrasse 100, 8091, Zurich Switzerland.; 15 Dermatology Department, Aristotle University, School of Medicine, Thessaloniki, Greece.; 16 Oncology Department, ``Bioclinic`` Private Hospital, Thessaloniki, Greece.; 17 Department of Food Science and Technology, International Hellenic University, Thessaloniki, Greece.; 18 Surgery Department, ``Genesis`` Private Hospital, Thessaloniki, Greece.; 19 Oncology Department, General Hospital of Rhodes, Greece.; 20 Department of Surgery, University Hospital of Alexandroupolis, Medical School, Democritus University of Thrace, Alexandroupolis, Greece.; 21 Ear, Nose and Throat (ENT) Department, Ludwig-Maximilians University of Munich, Munich, Germany.

**Keywords:** EBUS, non-small cell lung cancer, chemotherapy, immunotherapy, nivolumab, pembrolizumab, cisplatin.

## Abstract

**Introduction:** Immunotherapy is being used for the past five years either as first line or second line treatment with great results. Chemotherapy and radiotherapy have been also used as combination to immunotherapy to further enhance this type of treatment. Intratumoral treatment has been previously proposed as a treatment option for certain non-small cell lung cancer patients.

**Patients and Methods:** We recruited in total seventy four patients with non-small cell lung cancer in their second line treatment who received only chemotherapy in their first line treatment with programmed death-ligand-1 ≤ 50. Only adenocarcinoma or squamous cell carcinoma, and all negative for epidermal growth factor receptor, anaplastic lymphoma kinase, proto-oncogene tyrosine-protein kinase-1 and proto-oncogene B-Raf. Data were first examined with descriptive statistics choosing frequencies for categorical variables and histograms for the continuous ones. Twenty five received only intravenous immunotherapy and forty-nine intravenous cisplatin with immunotherapy. Data were first examined with descriptive statistics choosing frequencies for categorical variables and histograms for the continuous ones.

**Results:** The relationships between changes of performance status and disease progression were examined via a single correspondence analysis. The two-dimensional scores (coordinates) derived from the correspondence analysis were then regressed against the predictors to form distinct splits and nodes obtaining quantitative results. The best fit is usually achieved by lowering exhaustively the AICc criterion and looking in parallel the change of R^2^ expecting improvements more than 5%. both types of therapy are capable of producing best ameliorative effects, when either the programmed death-ligand-1 expression or parenchymal site in joint with low pack years are present in the sampling data.

**Conclusions:** Intratumoral treatment combination with cisplatin plus immunotherapy indifferent of nivolumab or pembrolizumab combination is an effective choice. In specific for those with endobronchial lesions. Moreover; patients with programmed death-ligand-1 ≥ 50 had their performance status and disease progression improved over the eight month observation.

## Introduction

Non-small cell lung cancer (NSCLC) is diagnosed at a late due to early disease symptoms. Therefore lung cancer screening has been proposed for early disease diagnosis 1, 2. We have several different techniques and diagnostic tools for lung cancer diagnosis such as; ct guided biopsies, ultrasound transthoracic biopsies, endobronchial ultrasound EBUS-convex transbrochial biopsy needle aspiration (EBUS-TBNA), radial EBUS 3-6. New novel navigation systems are also being used 7, 8. After the histology identification a molecular investigation is performed to the tissue sample in order to identify the proper treatment in advanced stage NSCLC patients. Regarding adenocarcinoma we investigate for epidermal growth factor receptor (EGFR), anaplastic lymphoma kinase (ALK), proto-oncogene tyrosine-protein kinase-1 (ROS), proto-oncogene B-Raf (BRAF) and programmed death-ligand 1 (PD-L1) 9-11. Regarding squamus cell carcinoma we investigate the expression of programmed death-ligand 1 (PD-L1). In the case were EGFR, ALK, BRAF or ROS-1 is identified we provide tyrosine kinase inhibitors (TKIs) as treatment 12. In the case of PD-L1 expression, based on the value ≥ or ≤ we administer only immunotherapy or a combination of immunotherapy plus chemotherapy 12. Regarding none other specified (NOS) we can still perform a molecular panel. Chemotherapy along with anti-angiogenic drugs or radiotherapy is also administered in the case where no gene expression is identified. Intratumoral treatment administration has been previously proposed and administered in advanced stage patients in order to shrink a large tumor that blocks an airway or where systemic therapy was not possible 13-19. It has been previously investigated that the higher the expression of PD-L1 the more efficient is the immunotherapy 20. Although there are patients with zero PD-L1 expression where immunotherapy is still effective for them 20. Moreover; it has been observed that radiotherapy and chemotherapy prior to immunotherapy enhances immunotherapy treatment results 20-23. In our current study we investigated the effectiveness of cisplatin administration with nivolumab or pembrolizumab in endobronchial or parenchymal lung cancer masses. The concept was to apply intratumoraly immunotherapy with cisplatin in order to enhance the treatment effect along with intravenous treatment.

## Patients and Methods

### Patients

**Figure 1:**
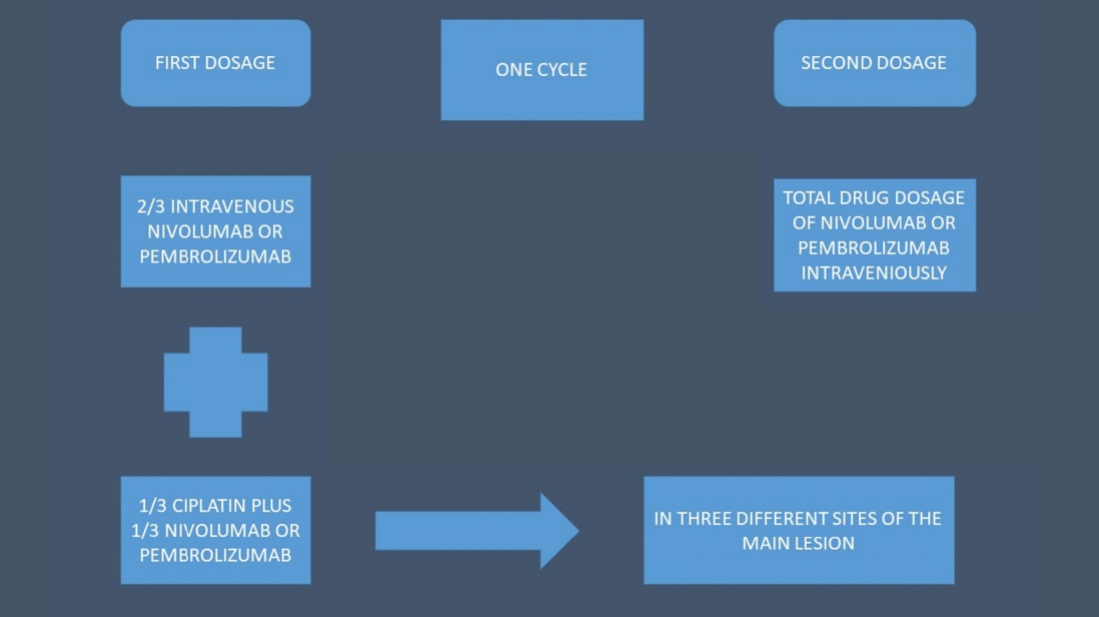
Schematic of the treatment structure.

We recruited seventy four non-small cell lung cancer (NSCLC) patients stage IV with either central endobronchial mass or lung parenchymal mass of ≥ 4-8 cm in maximum diameter. All patients were diagnosed with adenocarcinoma or squamus cell carcinoma. All patients were negative for EGFR, ALK, ROS-1 and BRAF. PD-L1 was investigated in all patients with DAKO technique. All patients had performance status (PS) 0-2 and were separated in two groups: 1) intravenous administration of immunotherapy with nivolumab or pembrolizumab according to the instructions of the drug regulation approval, 2) intratumoral group received cisplatin with a 19G needle (Olympus®) the 1/3 of the normal intravenous dosage (range 158-250mg) and the 1/3 the normal immunotherapy drug dosage (either nivolumab or pembrolizumab). The rest of their drug dosage of immunotherapy either nivolumab or pembrolizumab was administered itraveniously (i.v). The intratumoral administration was performed one time in every cycle (Figure 1).

### Methods

**Figure 2:**
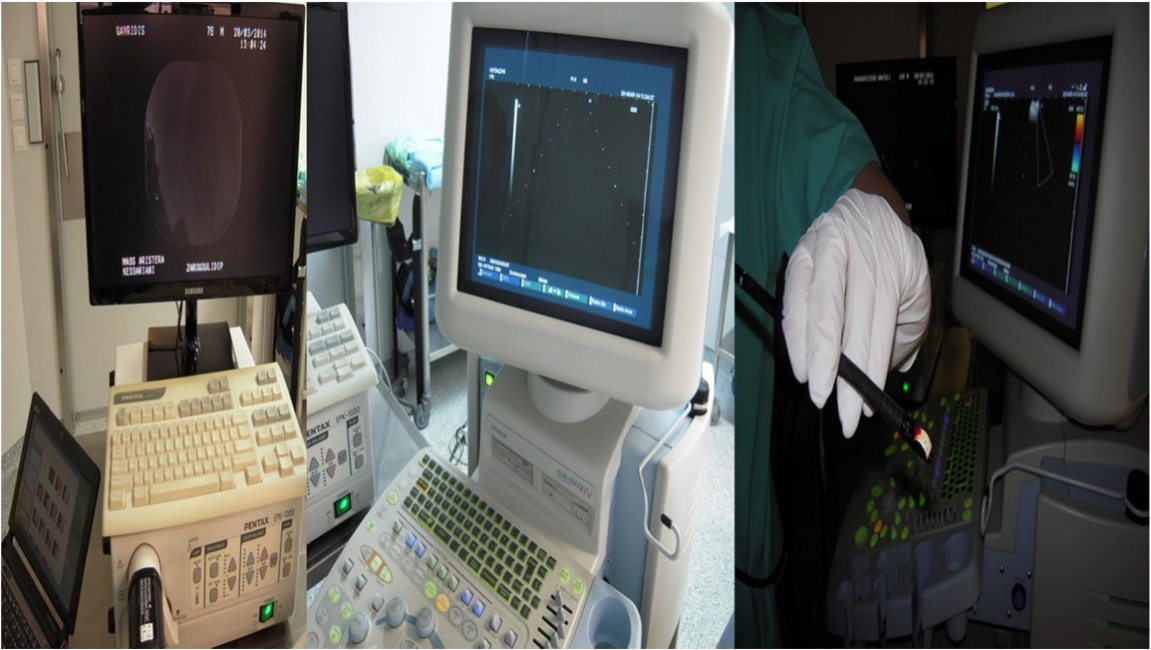
From left to right: PENTAX videoprocessor EPK-1000, middle; HITACHI EUB-7000EB, right; PENTAX EB-1970UK CONVEX ENDOSCOPE.

A PENTAX EB-1970UK EBUS convex endoscope was used with a PENTAX videoprocessor EPK-1000 and a HITACHI 7000EUB ultrasound power were used as equipment (Figure 2).

**Figure 3:**
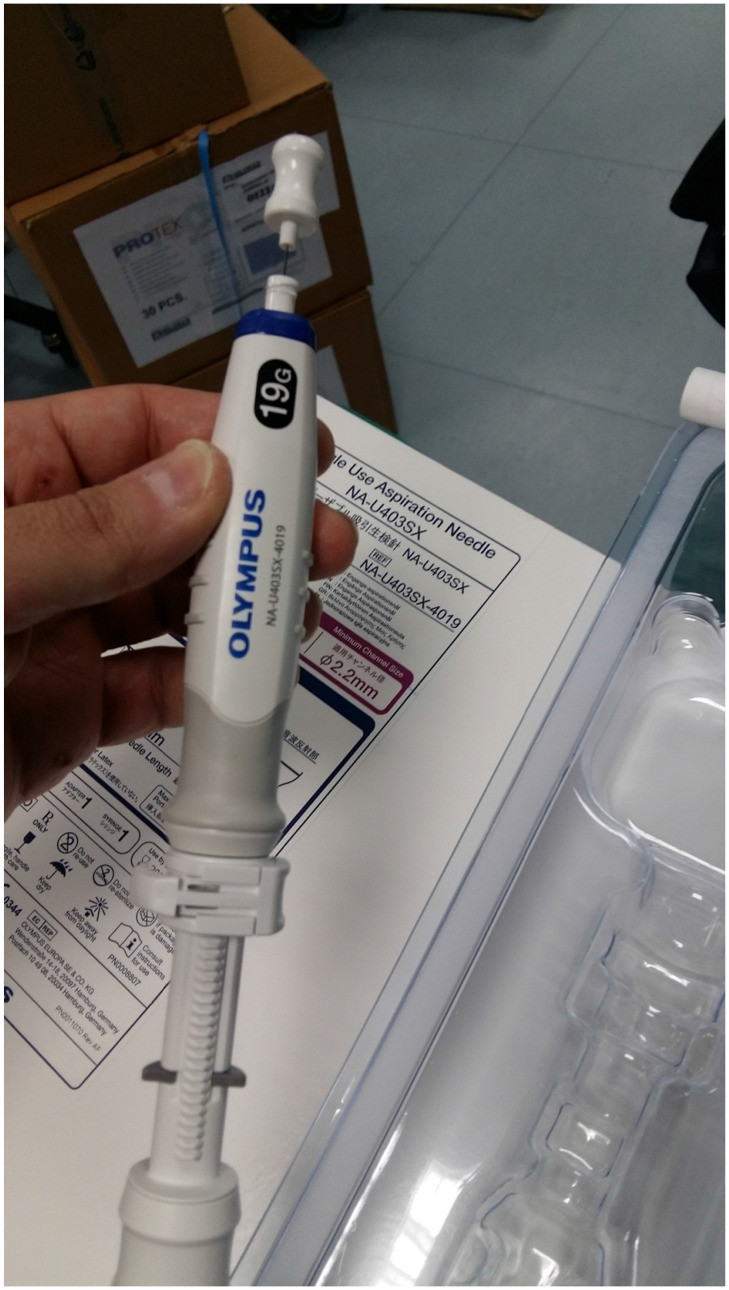
Olympus® 19G needle.

The needle that we used to puncture the endobronchial or parenchymal mass was an Olympus® 19G (Figure 3).

**Figure 4:**
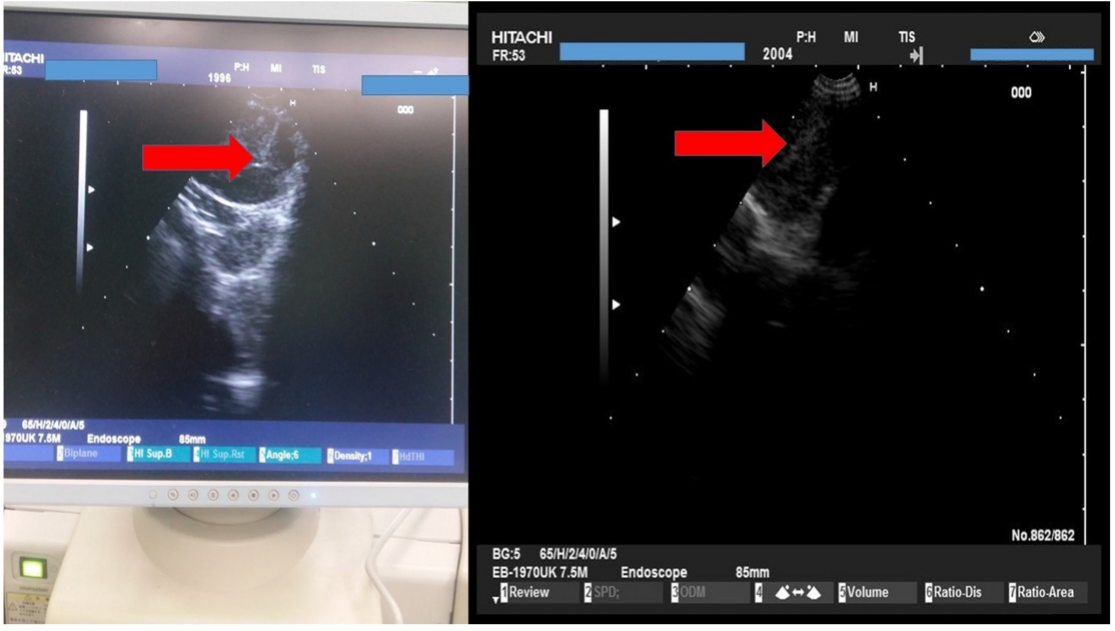
HITACHI EUB-7000EB images from different patients during administration.

We used the tip of the convex probe to visualize the lesion and we punctured the mass and injected the two drugs. Firstly cisplatin was applied and then nivolumab or pembrolizumab in three different sections of the lesion. We administered the immunotherapy drugs after cisplatin in every puncture, by doing this we did less punctures and we induced higher local treatment efficiency with the combination (Figure 4).

**Figure 5:**
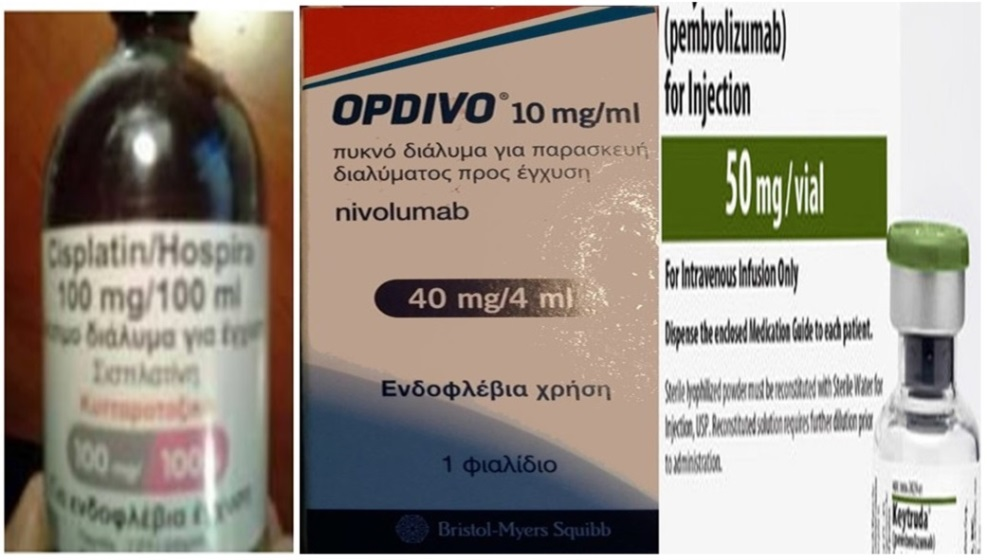
From left to right: non-specific cytotoxic agent cisplatin/hospira 100mg/100ml, ONCO-TAIN^™^, HOSPIRA UK, LIMITED. The immunotherapy drugs were Opdivo^®^ (nivolumab), Bristol-Myers Squibb, 10mg/l and Keytruda^®^ (pembrolizumab), Merck.

The cisplatin formulation was non-specific cytotoxic agent cisplatin/hospira 100mg/100ml, ONCO-TAIN^™^, HOSPIRA UK, LIMITED. The immunotherapy drugs were Opdivo^®^ (nivolumab), Bristol-Myers Squibb, 10mg/l and Keytruda^®^ (pembrolizumab), Merck (Figure 5).

**Figure 6:**
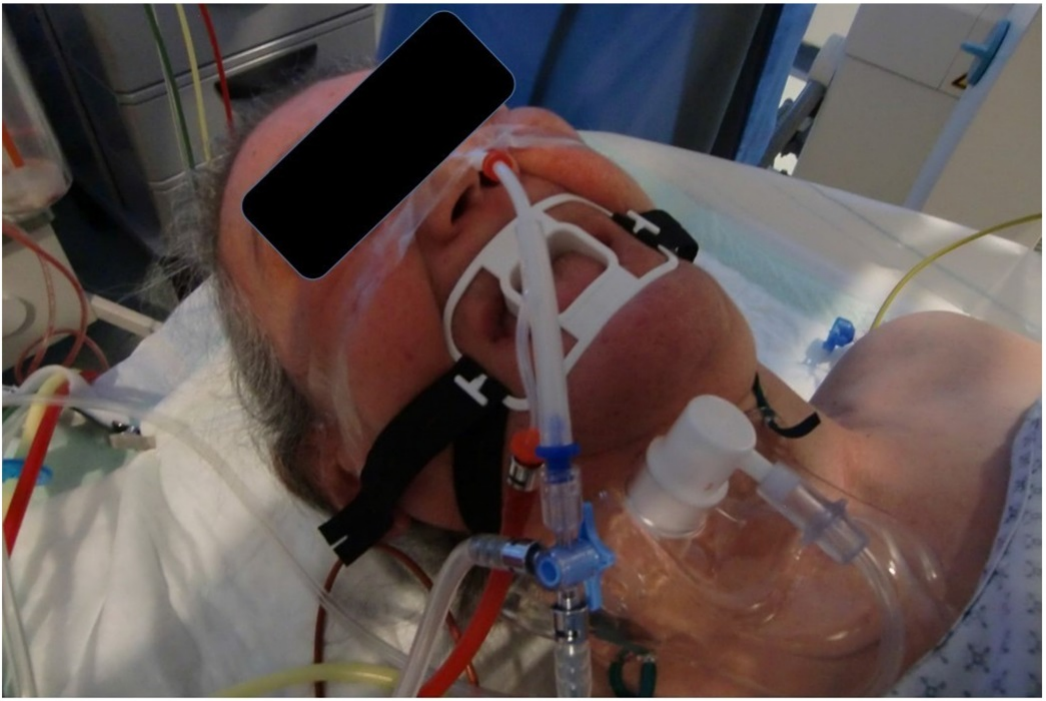
Patient during the administration procedure under jet-ventilation

All patients received mild anesthesia and were under jet-ventilation. The duration of each intratumoral administration was between 20-35 minutes (Figure 6).

No adverse effects were observed other than those observed in a diagnosed biopsy such mild hemoptysis. All patients included had a diagnosis of adenocarcinoma or squamus cell carcinoma none were non-other specific (NOS) or large cell. Also, all had previously been treated with chemotherapy with different combinations such as; cisplatin/carboplatin, paclitaxel/docetaxel, gemcitabine or navelbine. None had anti-angiogenic administration. All patients included were in second line treatment and the PD-L1 expression was ≤ 49. Their performance status (PS) was evaluated upon inclusion and re-evaluated every 4 months according to the immunotherapy criteria. The disease progression was evaluated upon inclusion and during re-staging with positron emission tomography according to the PERCIST criteria 24-26. The observation of the patient was stopped when the second progression of the disease was recorded.

### Statistical Materials and methods

Data were first examined with descriptive statistics choosing frequencies for categorical variables and histograms for the continuous ones. Performance status (PS) was recorded in increasing order of deterioration grade (0, 1, 2) 27 and the disease progress (PG) in increasing order of health improvement (1, 2, 3, 4) in accordance with the outcomes1 progression disease (PD), 2 stable disease (SD), 3 partial response (PR) and 4 complete response (CR). Patients were monitored 0, 4 and 8 months for PS and 4 and 8 months for PG. To simplify the monitoring for the sequential months a three-digit concatenation was formed for PS, e.g. 001 denotes **change PS** only in month 8 and 210 denotes gradual improvement till month 8, and a two-digit concatenation for PG, e.g. 23 denotes an outcome **change PG** from SD to PR.

As the main interest of the study is to describe outcomes from symptoms, first a single correspondence analysis was conducted including only the table of the cross-tabulated change PS with change PG, aiming to find clusters of points (categories) with particular attributes 28. The first two ``dimensions`` derived from the correspondence analysis were further analysed as responses against all the variables under study employing the regression classification trees 29. This technique fits the mean response values that are produced by portioning the variables (predictors) into particular splits and nodes maximizing each time the difference in the mean responses between the nodes of the splits. Continuous predictors are split by cutting values and categorical predictors are simply divided in two groups of levels. High determined R^2^ values and low AICc (Akaike Information Criterion) values promise for an adequate model, enhanced also by a cross-validation using part of data and the logworth criterion of splitting which should be greater than 1.3 (or equivalently less than 0.05).

## Results

In the sample of patients concerning categorical parameters (Table 1), higher contributions are met for squamus (62.2%), parenchymal area (71.6%) and approximately equal for tumor sizes (4-6 cm and ≥6 cm). The intratumoral injection was administered in a ratio of 2:1 against the intravenious (50:24, 67.6:32.4%) and the therapy treatment included simply immunotherapy (nivolumab and pembrolizumab) plus cisplatin as a combined treatment.

**Figure 7:**
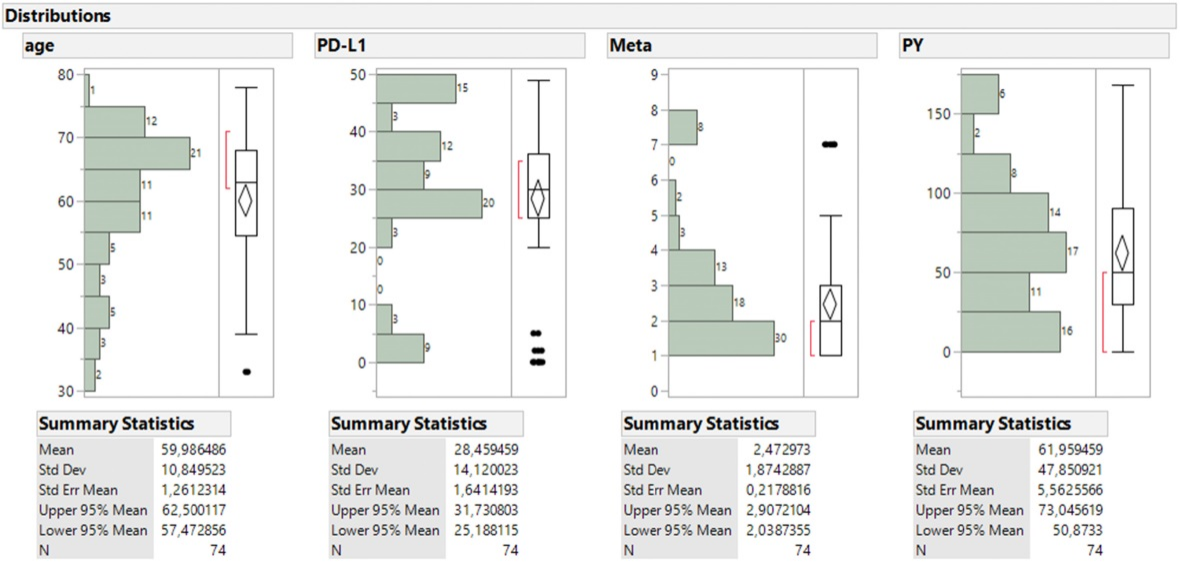
Size frequency distribution of the four continuous parameters accompanied with box plot and summary statistics.

For the continuous parameters (Figure 7), patients aged mostly 55 to 75 y.o. and comprised the 74% of sample with a mean value of 60 y.o. and the bulk of metastatic sites ranged between 1 to 4 (51%). The majority of PDL-1 expression was found above the 25% level (79.7%), non-smokers were numbered 15, smokers below 50 PY regard 12 patients, smokers between 50 and 100 PY regard 31 smokers and 12 found with more than 100 PY. One should keep in mind in order to comprehend the statistical analysis without being a statistician that the values for the performance status are explained as follows 0 good biological condition, 1 mild biological condition, 2 very bad biological condition.

Performance status and disease progression were cross-tabulated incorporating also the periodical changes (Table 2).

Sixteen patients had a stable good performance status (000), consisting mainly from change SD to PR (10 patients) and surprisingly from the inverse condition (4 patients). The improvement of patient's condition results from the changes PS 100 and 110 (worse biological condition from 1 to 0) including 10 patients and reflecting changes PG from 33 to 44 (partial response to complete response see above). A steady mild performance (111 performance status stable to biological condition 1) was noted in change PG 23 (8 patients from stable disease to partial response) totalling across the row 14 patients. The most striking drastic effect of improvement occurs exclusively in the combined cell of PS 210 (biological condiation from 2 (worse) to 0 (very good) and PG 34 (8 patients partial response to complete response). Bad performance (2) status in joint with bad outcome happens in the low left corner of the table in which changes PS 221 and 222 keep up with changes PG 11 (progression disease) and 22 (13 patients stable disease). The most highlights of that table reveal a successive deterioration by time for the performance status of 001 and 112, summing up 7 patients and a successive amelioration by time for PS 100, 110, 210 and 221 totalling 28 patients. Of those, 24 patients reach finally the performance 0 and 4 pass in the intermediate stage (221).

**Figure 8:**
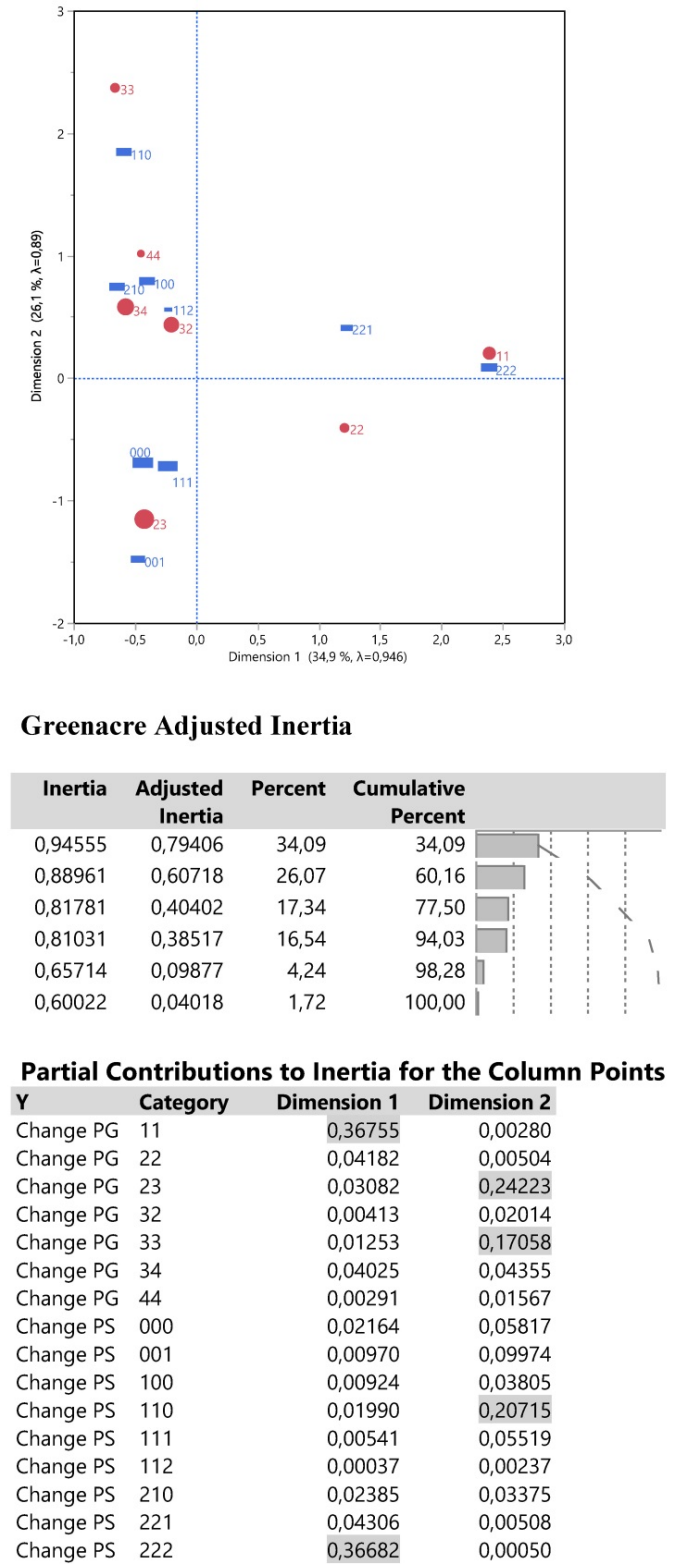
Output from a two dimensional single correspondence analysis with changes PS and PG including the correspondence plot, the adjusted inertia and the individual contribution of the parameter categories. The size of red points reflects the frequency distribution of change PG from **Table 2**.

The relationships between changes PS and PG were examined via a single correspondence analysis whose results are shown in Figure 8.

The Grenacre adjusted inertia highlighted 60.2% of the total variation including the first two dimensions (an analogue to major principal components), whose corresponding plot of joint points shows the following: in the right half part, the complete bad performance (221and 222) and bad disease progression (11 and 22) is located uniquely around the dimension 1, so describing it very effectively since both PS and PG changes contribute 73.4% (26.7% each) of the first inertia. In the left half part, an increasing order of best outcome changes PG is arrayed from bottom to top, that is from 23 to 34, 44 and 33. High contributions to inertia 2 are performed by changes PG 23 and 33 (24.2% and 17.0%), that is the extreme points of dimension 2 and by change PS 110 (20.1%) which is poisoned close to change PG 33. In other words, values of direction1 greater than 1 signal for bad patients' condition and values for direction 2 either lower than -0.5 or greater than 1 signify for health amelioration of the disease progression.

The two-dimensional scores (in standard deviations) derived from the correspondence analysis were then regressed against the predictors to form distinct splits and nodes obtaining quantitative results. The best fit is usually achieved by lowering exhaustively the AICc criterion and looking in parallel the change of R^2^ expecting improvements more than 5%.

**Figure 9:**
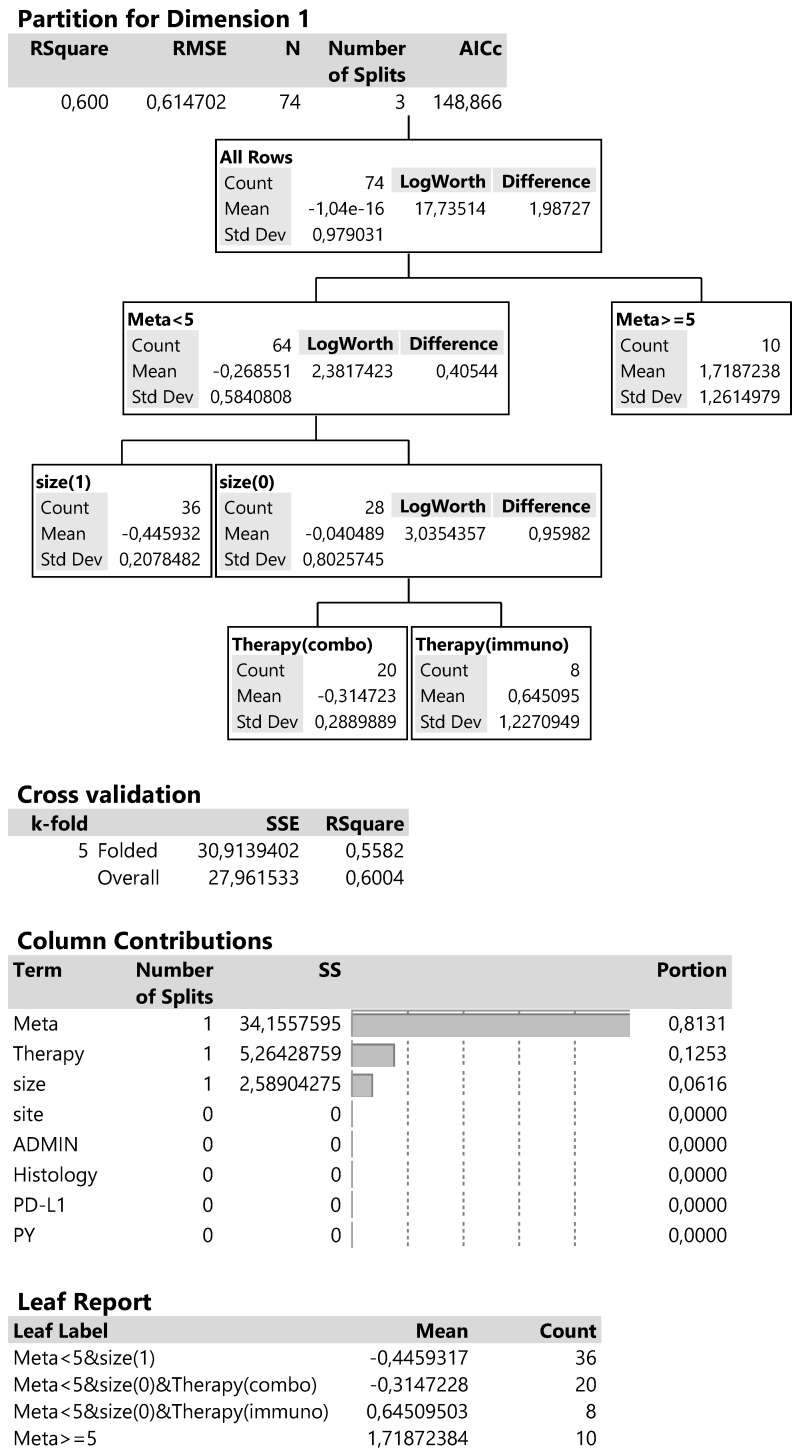
Decision trees for the corresponding dimension 1 according to regressive classification of predictors.

Dimension 1 is best described by a 60% R^2^ and AICc criterion equal to 148.9 (Figure 9) and includes the following: the initial division splits the metastatic sites below and above 5, then at meta <5 (more than 1 metastatic site) splits the tumor sizes and finally the type of therapy at the smaller tumor size (4-6cm). The contributions of the above predictors in the whole regression tree reach 81.3% for the metastatic sites, 12.5% for therapy type and 6.2% for tumor size. The cross validation or 5 parts of data produces a 55.8% R^2^ close to the 60% of the analysis so suggesting a good partition of predictors.

In summary, the combined effects of predictors in the leaf report, show a high and positive mean value of dimension 1 scores (1.72), indicative for patients with metastatic sites more than 5 (more than 1 metastatic site) and corresponding to the bad changes PS and PG. Patients with metastatic sites less than 5, smaller tumor size (4-6cm) and treated with immunotherapy manifest a lower bad condition (mean score 0.64). On the other hand, the negative dimension scores propose patients with good performance and outcome (see also the correspondence plot) and that is achieved at lower metastatic sites (<5), tumor size of 4-6cm and therapy with the combined treatment (mean score -0.31) and even better with low metastatic sites and tumor size >6cm (mean score -0.44).

Dimension 2 is best described by a 43.6% R^2^ and 174.6 AICc criterion, with the formation of 5 splits (Figure 9).

The first division starts with therapy split, then continues with the PD-L1 split and sites of infection at opposite nodes and terminates with tumor size and packs per year (PY) again at opposite nodes. The cross-validation with 10 parts of data approaches a 30.4% R^2^ as compared to 43,6% revealing a relatively reliable classification. The % contribution of the five predictors is shown is decreasing order of magnitude: PD-L1, PY, therapy treatment, site and size, descending from 28.6% to 12.3%.

Consulting the leaf report and the left part of divisions, it appears that immunotherapy in connection with PD-L1 ≥25% and tumor size ≥ 6cm is clearly described in the correspondence plot by the change PG 23 and a sequential trend PS 001 (mean score -1.06). When the small tumor size is present instead, the effect becomes negligible (mean score -0.19). Best outcome PG and performance status PS distinctly appears when immunotherapy is connected with PD-L1 ≤25% (mean score 0.74, changes PG 34 and 44, performance PS 210 and 100). At the right part of divisions, the combined therapy together with parenchymal sites and PY ≤30 manifest an area in the correspondence plot similar to that explained in the left part of divisions (mean score 0.76). Thus, two alternative combinations of predictors lead to an equal effect. A roughly similar approach is evident when the combined treatment is affiliated with endobronchial sites (mean score 0.63).

In conclusion, for dimension 2 both types of therapy are capable of producing best ameliorative effects, when either the PD-L1 expression or parenchymal site in joint with low PY are present in the sampling data. For dimension 1, the prognosis for patients with more than 5 metastatic sites is discouraging although treating with immunotherapy those with smaller tumors could drive to less compounding effects.

## Discussion

Non-small cell lung cancer patients are usually diagnosed at a late stage and systemic drugs are usually administered. According to the genome of the patients we have chemotherapy treatment, tyrosine kinase inhibitors and immunotherapy. Intratumoral chemotherapy and combination of this treatment modality have been investigated in the past twenty years both in animal models and patients 13, 15, 16, 30-33. There are several issues to address before starting such a study. Firstly we chose the study population in order to have the same genome of lung cancer we chose only adenocarcinoma or squamous cell carcinoma as this choice plays a major role in the response. We included only patients with PD-L1 expression ≤ 50 in order to evaluate the effect of the combination therapy with cisplatin. As it has been previously observed the addition of chemotherapy locally increases the distraction of the tumor and the increased antigens produced locally enhance the effect of immunotherapy 34. The same has been observed with radiotherapy 35-37. With local intratumoral therapy less adverse effects have been observed 38, 39. Moreover; the issue method of administration, should be easy for the patient and without complication. EBUS-TBNA is a safe method under mild anesthesia and with small needles that do not cause any serious adverse effects 4, 5, 40, 41. We had to carefully choose which patients would benefit from this treatment modality and these patients would be two groups with endobronchial lesions and large parenchymal masses. Indeed this was confirmed in our study. In specific patients with PD-L1 expression ≥50 and endobronchial lesion of ≥4cm had their performance status improved along with their disease status over the 8 months of observation. Furthermore; patients with PD-L1 expression ≥50 and parenchymal lesions ≤8cm had also their performance status improved along with their disease. We had included also patients with 0 PD-L1 expression which benefited from the combination intratumoral treatment with their performance status improved along with their disease indifferent of an endobronchial or parenchymal lesion. This is known from the first studies of immunotherapy and it is explained with unknown mechanisms of immunoitherapy 42. Patients with multiple metastatic site and increased tumor burden did not benefit from the combo intratumoral as these patients need systemic drug release. The intratumoral combination therapy is an efficient method of administration, and it could be a chose for specific patients such as those with obstructive endobronchial disease.

## Figures and Tables

**Table 1:** Numerical and percentage frequency distribution of categorical parameters

	Sex		Size		Therapy		ADMIN		Histology		Site	
	0	1	0	1	combo	immuno	0	1	1	2	0	1
N	34	40	35	39	50	24	24	50	28	46	21	53
Row %	45,95%	54,05%	47,30%	52,70%	67,57%	32,43%	32,43%	67,57%	37,84%	62,16%	28,38%	71,62%

**Table 2:** Cross tabulated frequencies between change performance status (PS) and change disease progression (PG).

	Change PG							
**Change PS**	**11**	**22**	**23**	**32**	**33**	**34**	**44**	**All**
**000**	0	0	10	4	0	2	0	16
**001**	0	0	6	0	0	0	0	6
**100**	0	0	0	4	0	2	2	8
**110**	0	0	0	2	4	2	0	8
**111**	0	2	8	1	0	3	0	14
**112**	0	0	0	1	0	0	0	1
**210**	0	0	0	0	0	8	0	8
**221**	2	0	0	2	0	0	0	4
**222**	7	2	0	0	0	0	0	9
**All**	9	4	24	14	4	17	2	74

## References

[1] Patz EF Jr, Caporaso NE, Dubinett SM, Massion PP, Hirsch FR, Minna JD (2010). National Lung Cancer Screening Trial American College of Radiology Imaging Network Specimen Biorepository originating from the Contemporary Screening for the Detection of Lung Cancer Trial (NLST, ACRIN 6654): design, intent, and availability of specimens for validation of lung cancer biomarkers. Journal of thoracic oncology: official publication of the International Association for the Study of Lung Cancer.

[2] Ostrin EJ, Sidransky D, Spira A, Hanash SM (2020). Biomarkers for lung cancer screening and detection. Cancer epidemiology, biomarkers & prevention: a publication of the American Association for Cancer Research, cosponsored by the American Society of Preventive Oncology.

[3] Zaric B, Stojsic V, Sarcev T, Stojanovic G, Carapic V, Perin B (2013). Advanced bronchoscopic techniques in diagnosis and staging of lung cancer. Journal of thoracic disease.

[4] Zarogoulidis P, Petridis D, Sapalidis K, Tsakiridis K, Baka S, Vagionas A (2020). Lung cancer biopsies: Comparison between simple 22G, 22G upgraded and 21G needle for EBUS-TBNA. Journal of Cancer.

[5] Zaric B, Stojsic V, Carapic V, Kovacevic T, Stojanovic G, Panjkovic M (2016). Radial Endobronchial Ultrasound (EBUS) Guided Suction Catheter-Biopsy in Histological Diagnosis of Peripheral Pulmonary Lesions. Journal of Cancer.

[6] Oezkan F, Khan A, Zarogoulidis P, Hohenforst-Schmidt W, Theegarten D, Yasufuku K (2014). Efficient utilization of EBUS-TBNA samples for both diagnosis and molecular analyses. OncoTargets and therapy.

[7] Li S, Yan W, Chen M, Li Z, Zhu Y, Wu Q (2020). Virtual bronchoscopic navigation without fluoroscopy guidance for peripheral pulmonary lesions in inexperienced pulmonologist. Chinese journal of cancer research = Chung-kuo yen cheng yen chiu.

[8] Ma L, Fang Y, Zhang T, Xue P, Bo L, Liu W (2020). Comparison in efficacy and safety of forceps biopsy for peripheral lung lesions guided by endobronchial ultrasound-guided sheath (EBUS-GS) and electromagnetic navigation bronchoscopy combined with EBUS (ENB-EBUS). American journal of translational research.

[9] Tsoulos N, Papadopoulou E, Metaxa-Mariatou V, Tsaousis G, Efstathiadou C, Tounta G (2017). Tumor molecular profiling of NSCLC patients using next generation sequencing. Oncology reports.

[10] Domvri K, Zarogoulidis P, Darwiche K, Browning RF, Li Q, Turner JF (2013). Molecular Targeted Drugs and Biomarkers in NSCLC, the Evolving Role of Individualized Therapy. Journal of Cancer.

[11] Domvri K, Darwiche K, Zarogoulidis P, Zarogoulidis K (2013). Following the crumbs: from tissue samples, to pharmacogenomics, to NSCLC therapy. Translational lung cancer research.

[12] Zarogoulidis K, Zarogoulidis P, Darwiche K, Boutsikou E, Machairiotis N, Tsakiridis K (2013). Treatment of non-small cell lung cancer (NSCLC). Journal of thoracic disease.

[13] Hohenforst-Schmidt W, Zarogoulidis P, Darwiche K, Vogl T, Goldberg EP, Huang H (2013). Intratumoral chemotherapy for lung cancer: re-challenge current targeted therapies. Drug design, development and therapy.

[14] Hohenforst-Schmidt W, Zarogoulidis P, Stopek J, Kosmidis E, Vogl T, Linsmeier B (2015). Enhancement of Intratumoral Chemotherapy with Cisplatin with or without Microwave Ablation and Lipiodol. Future Concept for Local Treatment in Lung Cancer. Journal of Cancer.

[15] Baliaka A, Zarogoulidis P, Domvri K, Hohenforst-Schmidt W, Sakkas A, Huang H (2014). Intratumoral gene therapy versus intravenous gene therapy for distant metastasis control with 2-diethylaminoethyl-dextran methyl methacrylate copolymer non-viral vector-p53. Gene therapy.

[16] Celikoglu F, Celikoglu SI, York AM, Goldberg EP (2006). Intratumoral administration of cisplatin through a bronchoscope followed by irradiation for treatment of inoperable non-small cell obstructive lung cancer. Lung cancer.

[17] Feng W, Li J, Han S, Tang J, Yao J, Cui Y (2016). [CT Guided Radiofrequency Ablation Followed Intratumoral Chemotherapy in the Treatment of Early Stage Non-small Cell Lung Cancer]. Zhongguo fei ai za zhi = Chinese journal of lung cancer.

[18] Jiang W, Yang X, Wang X, Li Y, Yang X, Wang N (2020). Bronchoscopic intratumoral injections of cisplatin and endostar as concomitants of standard chemotherapy to treat malignant central airway obstruction. Postgraduate medical journal.

[19] DeMaio A, Sterman D (2020). Bronchoscopic intratumoural therapies for non-small cell lung cancer. European respiratory review: an official journal of the European Respiratory Society.

[20] Guven DC, Sahin TK, Dizdar O, Kilickap S (2020). Predictive biomarkers for immunotherapy efficacy in non-small-cell lung cancer: current status and future perspectives. Biomarkers in medicine.

[21] Samuel E, Lie G, Balasubramanian A, Hiong A, So Y, Voskoboynik M (2020). Impact of Radiotherapy on the Efficacy and Toxicity of anti-PD-1 Inhibitors in Metastatic NSCLC. Clinical lung cancer.

[22] Awad MM, Gadgeel SM, Borghaei H, Patnaik A, Yang JC, Powell SF (2020). Long-Term Overall Survival From KEYNOTE-021 Cohort G: Pemetrexed and Carboplatin With or Without Pembrolizumab as First-Line Therapy for Advanced Nonsquamous NSCLC. Journal of thoracic oncology: official publication of the International Association for the Study of Lung Cancer.

[23] Borghaei H, Langer CJ, Gadgeel S, Papadimitrakopoulou VA, Patnaik A, Powell SF (2019). 24-Month Overall Survival from KEYNOTE-021 Cohort G: Pemetrexed and Carboplatin with or without Pembrolizumab as First-Line Therapy for Advanced Nonsquamous Non-Small Cell Lung Cancer. Journal of thoracic oncology: official publication of the International Association for the Study of Lung Cancer.

[24] Garcia-Perez FO, Medina-Ornelas SS, Barron-Barron F, Arrieta-Rodriguez O (2020). Evaluation of non-small cell lung cancer by PET/CT with (64)CuCl2: initial experience in humans. American journal of nuclear medicine and molecular imaging.

[25] Wu Q, Liu J, Zhang Y, Wu S, Xie X (2020). Predictive value of positron emission tomography for the prognosis of immune checkpoint inhibitors (ICIs) in malignant tumors. Cancer immunology, immunotherapy: CII.

[26] Rossi G, Bauckneht M, Genova C, Rijavec E, Biello F, Mennella S (2020). Comparison Between (18)F-FDG PET-Based and CT-Based Criteria in Non-Small Cell Lung Cancer Patients Treated with Nivolumab. Journal of nuclear medicine: official publication, Society of Nuclear Medicine.

[27] Scott JM, Stene G, Edvardsen E, Jones LW (2020). Performance Status in Cancer: Not Broken, But Time for an Upgrade?. Journal of clinical oncology: official journal of the American Society of Clinical Oncology.

[28] M G (2017). Correspondence analysis in practice. 3rd ed. London: Chapman and Hall/CRC Press.

[29] Breiman L, Friedman J.H, Olshen R (1998). and Stone C. Classification and regression trees. London: Chapman and Hall/CRC Press.

[30] Hohenforst-Schmidt W, Zarogoulidis P, Stopek J, Vogl T, Hubner F, Turner JF (2015). DDMC-p53 gene therapy with or without cisplatin and microwave ablation. OncoTargets and therapy.

[31] Sakkas A, Zarogoulidis P, Domvri K, Hohenforst-Schmidt W, Bougiouklis D, Kakolyris S (2014). Safety and efficacy of suicide gene therapy with adenosine deaminase 5-fluorocytosine silmutaneously in *in vitro* cultures of melanoma and retinal cell lines. Journal of Cancer.

[32] Zarogoulidis P, Darwiche K, Sakkas A, Yarmus L, Huang H, Li Q (2013). Suicide Gene Therapy for Cancer - Current Strategies. Journal of genetic syndromes & gene therapy.

[33] Zarogoulidis P, Pavlioglou P, Pivert PL, Machairiotis N, Katsikogiannis N, Kougioumtzi I (2014). Current and future intratumoral targeted treatment for pancreatic cancer. Therapeutic delivery.

[34] Tong Z, Luo F, Yang X, Kang M, Lin J (2020). Platinum versus immunotherapy for early resectable non-small cell lung cancer: A protocol for systematic review and meta analysis. Medicine.

[35] Kroeze SGC, Fritz C, Schaule J, Siva S, Kahl KH, Sundahl N (2020). Stereotactic radiotherapy combined with immune- or targeted therapy for metastatic renal cell carcinoma. BJU international.

[36] Chicas-Sett R, Morales-Orue I, Castilla-Martinez J, Zafra-Martin J, Kannemann A, Blanco J (2019). Stereotactic Ablative Radiotherapy Combined with Immune Checkpoint Inhibitors Reboots the Immune Response Assisted by Immunotherapy in Metastatic Lung Cancer: A Systematic Review. International journal of molecular sciences.

[37] Moran A, Azghadi S, Maverakis EM, Christensen S, Dyer BA (2019). Combined Immune Checkpoint Blockade and Stereotactic Ablative Radiotherapy Can Stimulate Response to Immunotherapy in Metastatic Melanoma: A Case Report. Cureus.

[38] Gao Q, Tang S, Chen H, Chen H, Li X, Jiang Y (2020). Intratumoral injection of anlotinib hydrogel enhances antitumor effects and reduces toxicity in mouse model of lung cancer. Drug delivery.

[39] Baniel CC, Sumiec EG, Hank JA, Bates AM, Erbe AK, Pieper AA (2020). Intratumoral injection reduces toxicity and antibody-mediated neutralization of immunocytokine in a mouse melanoma model. Journal for immunotherapy of cancer.

[40] Sapalidis K, Zarogoulidis P, Petridis D, Kosmidis C, Fyntanidou B, Tsakiridis K (2019). EBUS-TNBA 22G samples: Comparison of PD-L1 expression between DAKO and BIOCARE((R)). Journal of Cancer.

[41] Zarogoulidis P, Huang H, Bai C, Kosmidis C, Porpodis K, Kallianos A (2018). A new mode of ventilation for interventional pulmonology. A case with EBUS-TBNA and debulking. Respiratory medicine case reports.

[42] Redman MW, Papadimitrakopoulou VA, Minichiello K, Hirsch FR, Mack PC, Schwartz LH (2020). Biomarker-driven therapies for previously treated squamous non-small-cell lung cancer (Lung-MAP SWOG S1400): a biomarker-driven master protocol. The Lancet Oncology.

